# Stomatocyte structural color-barcode micromotors for multiplex assays

**DOI:** 10.1093/nsr/nwz185

**Published:** 2019-11-21

**Authors:** Lijun Cai, Huan Wang, Yunru Yu, Feika Bian, Yu Wang, Keqing Shi, Fangfu Ye, Yuanjin Zhao

**Affiliations:** 1 Precision Medicine Center Laboratory, The First Affiliated Hospital of Wenzhou Medical University, Wenzhou 325035, China; 2 Wenzhou Institute, University of Chinese Academy of Sciences, Wenzhou 325001, China; 3 State Key Laboratory of Bioelectronics, School of Biological Science and Medical Engineering, Southeast University, Nanjing 210096, China; 4 Beijing National Laboratory for Condensed Matter Physics, Institute of Physics, Chinese Academy of Sciences, Beijing 100190, China

**Keywords:** structural color, micromotor, sensor, photonic crystal, multiplex assay

## Abstract

Artificial micromotors have a demonstrated value in the biomedical area. Attempts to develop this technology tend to impart micromotors with novel functions to improve the values. Herein, we present novel structural color-barcode micromotors for the multiplex assays. We found that, by rapidly extracting solvent and assembling monodispersed nanoparticles in droplets, it could form stomatocyte colloidal crystal clusters, which not only showed striking structural colors and characteristic reflection peaks due to their ordered nanoparticles arrangement, but also provided effective cavities for the integration of functional elements. Thus, the micromotors with catalysts or magnetic elements in their cavities, as well as with the corresponding structural color coding, could be achieved by using the platinum and ferric oxide dispersed pre-gel to fill and duplicate the stomatocyte colloidal crystal clusters. We have demonstrated that the self-movement of these structural color-barcode micromotors could efficiently accelerate the mixing speed of the detection sample and greatly increase the probe–target interactions towards faster and more sensitive single or multiplex detection, and the magnetism of these barcode micromotors enables the flexible collection of the micromotors, which could facilitate the detection processes. These features make the stomatocyte structural color-barcode micromotors ideal for biomedical applications.

## INTRODUCTION

Micromotors are artificial microscale devices that can achieve autonomous movement by converting supplied fuels or externally provided energy into kinetic energy [1–4]. Since the concept of micromotors was proposed, great scientific interest has been attracted; as a consequence, impressive progress has been made in exploiting various kinds of micromotors [5–7]. Micromotors in diverse shapes, including wires, rods, spheres and tubes, were proposed with different propulsion mechanisms, including bubble propulsion, self-electrophoresis, self-diffusiophoresis and self-acustophoresis [8–11]. Major efforts have revealed that micromotors present copious values in fields of biomedical engineering, environmental science and so on [12–14]. In particular, there is considerable interest in the use of micromotors in biosensing [15,16]. Compared with traditional methods, these micromotor-based strategies are able to greatly enhance the sensitivity while largely reducing the assay time of biomolecules, since the continuous movement of micromotors can increase the possibility of target–receptor contacts [17–20]. Although there has been much progress in this aspect, recently available micromotors employed in biosensors can only carry out single-target screening and cannot meet the requirement of multiplex and high-throughput analyses, which is usually necessary to guide diagnosis. Therefore, functional micromotors with multiplexing capabilities are still anticipated in biomedical areas.

**Figure 1.**
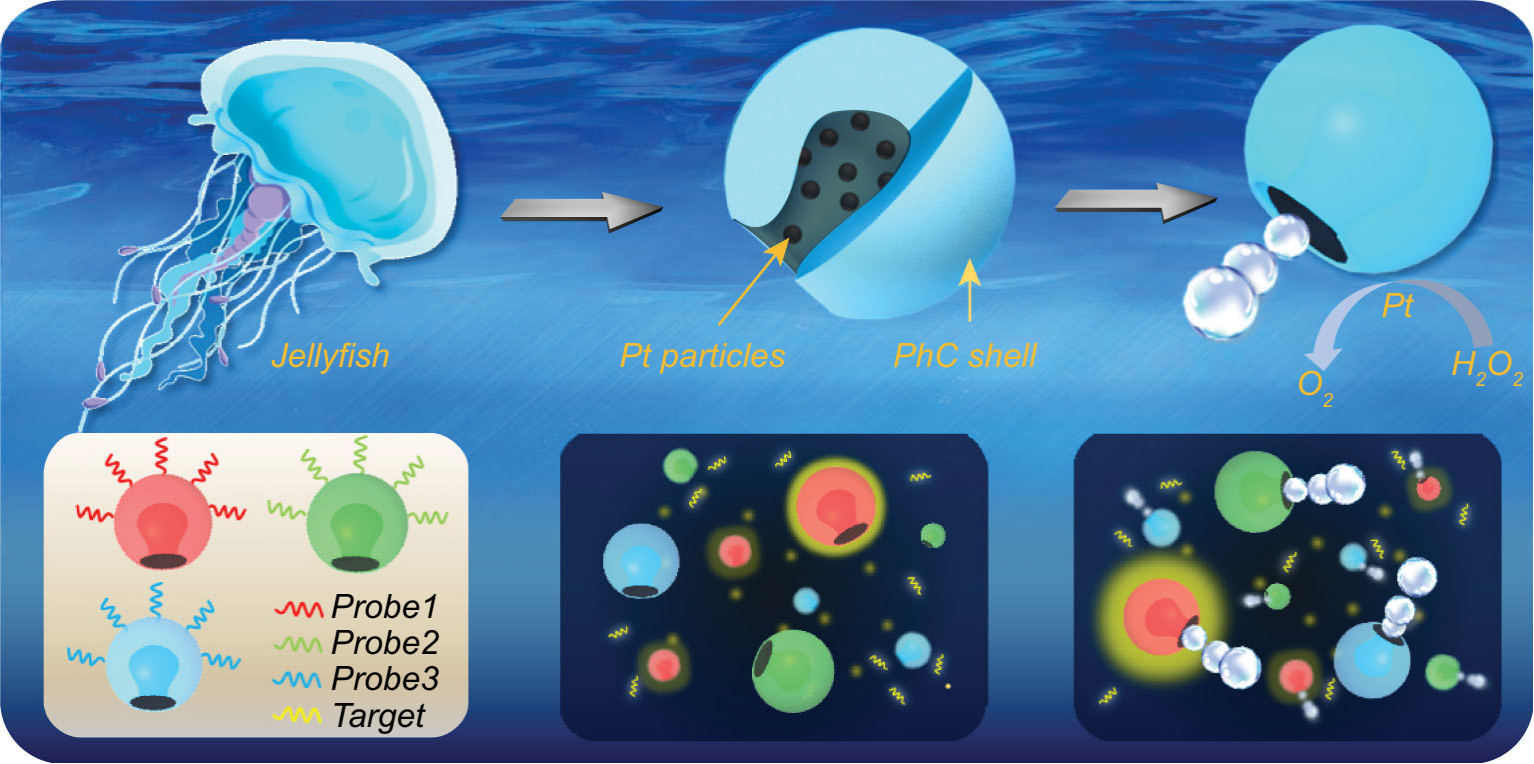
Scheme of the design of jellyfish-like structural color-barcode micromotors and their application in biosensing.

In this paper, we present novel structural color-barcode micromotors for multiplex assays, as schemed in Fig. 1. Barcodes, which encode information about their specific compositions and enable simple identification, have provided clues to realizing multiplex high-throughput bioassays [21,22]. Many encoding strategies including graphical, optical, electrical and biomolecular methods have been employed for the barcode particles [23–25]. Among these particles, photonic crystal (PhC)-derived structural color barcodes, whose codes are their characteristic reflection peaks originating from the photonic band gap (PBG), have attracted increasing interest [22,26,27]. As their peak positions, which are employed as the encoding elements, are based on their periodical physical structures, the structural color barcodes exhibit distinct advantages of remarkable encoding stability, freedom from any fluorescent background and photobleaching [28,29]. These properties make the structural color barcodes very convenient for practical applications [30–33]. However, because of the limitation of the structure of the encoded particles, these barcode technologies have never been integrated into self-driven micromotors, which will improve detection efficiency and sensitivity.

Herein, we found that particles of colloidal crystal clusters could form stomatocyte morphology by rapidly extracting solvent and assembling monodispersed nanoparticles into droplets. These stomatocyte particles could not only show striking structural colors and characteristic reflection peaks due to their ordered nanoparticles arrangement, but also provide effective cavities for the integration of functional elements. Thus, by using the platinum (Pt) and ferric oxide (Fe_3_O_4_) dispersed hydrogel to fill and duplicate the stomatocyte colloidal crystal particles, the barcode micromotors with catalyst or magnetic elements in their cavities, as well as with the corresponding structural color coding, could be achieved (Fig. 1). When these micromotors were exposed to a solution with hydrogen peroxide (H_2_O_2_) additive, the Pt-dispersed hydrogel in their cavities could propel the micromotors by expelling catalytic bubbles, while the present Fe_3_O_4_ could impart magnetic guidance for the micromotors. It was demonstrated that the self-movement of these structural color-barcode micromotors could efficiently accelerate the mixing speed of the detection sample and greatly increase the probe–target interactions towards faster and more sensitive detection; also, the magnetism of these barcode micromotors enables the flexible collection of the micromotors, which could facilitate the detection processes. Based on the specific identification feature of the stable structural color coding, the barcode micromotors could also perform an unprecedented simultaneous multiplexing capability for DNA detection. These results indicate that the structural color-barcode micromotors will provide an ideal platform for ultrasensitive multiplex assays in different fields.

## RESULTS AND DISCUSSION

**Figure 2.**
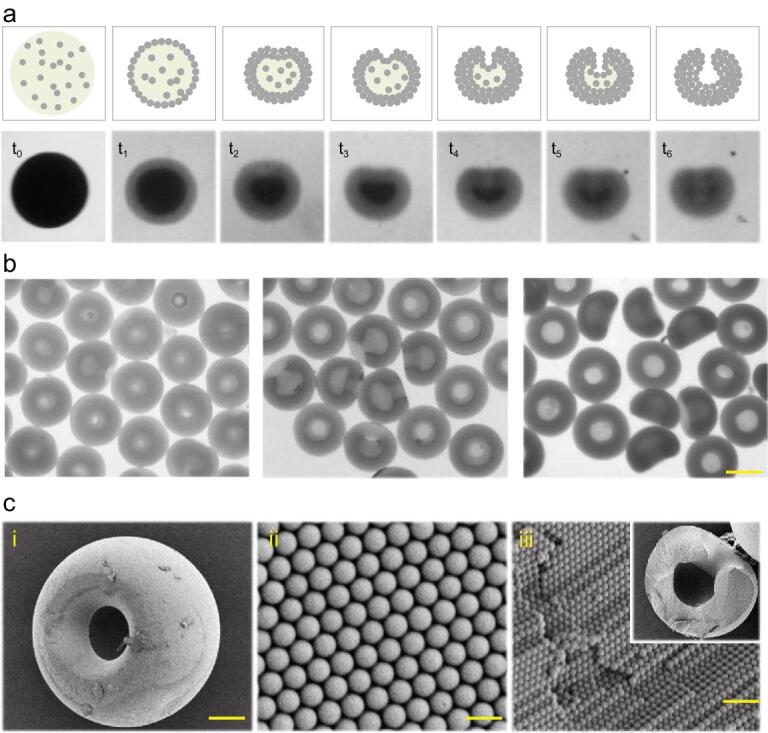
Fabrication of the stomatocyte PhC barcodes. (a) The schematic illustrations and bright-field microscopic images of the forming process of the stomatocyte PhC barcodes. (b) Bright-field microscopic images of the stomatocyte PhC barcodes with different aperture ratios. The scale bar is 180 μm. (c) SEM images of the (i) entire morphology, (ii) surface nanostructure and (iii) inner nanostructure of the stomatocyte PhC barcodes. The scale bars are 50 μm, 600 nm and 1 μm, respectively.

In a typical experiment, droplets containing monodispersed silica colloidal nanoparticles were exposed to organic extractant to remove the aqueous solvent for fast colloidal self-assembly. After this short period, the PhC particles dried from the droplets would present stomatocyte morphology as recorded and shown in Fig. 2a. First, the diameters of the droplets decreased rapidly as the solvent extracted and the circumferential nanoparticles of the droplets solidified. Due to the fast extraction rate, the interface of extraction moved much faster than the diffusion of the colloidal nanoparticles in the droplets and the concentration of colloidal nanoparticles at the interface was higher than that in the center, which led to the stretch and extrusion of the spherical droplets [34–37]. Thus, the top parts of the droplets began to fold inward at t_3._ After continuous folding inward until the assembly of the inner nanoparticles was completed, the stomatocyte PhC particles were finally formed. Because the formation was related to the extraction of solvent around the colloidal nanoparticles droplets, the morphology of the stomatocyte particles could be tailored by using different kinds of organic extraction agents and different concentrations of nanoparticles aqueous droplets. First of all, glyceryl triacetate, *n*-butyl alcohol and cyclopropylene glycol that had different solubilities were explored. Results showed that different organic solubilities resulted in different extraction rates, which could influence not only formation time, but also the morphology and order degree of the PhC particles (Supplementary Fig. S1). To be specific, the slower the nanoparticles assembled, the more orderly the particles were formed (Supplementary Fig. S2). In addition, stomatocyte PhC particles with different aperture ratios could be achieved by utilizing droplets with different concentration of nanoparticles (Fig. 2b). By increasing the concentration of the original droplets, the aperture ratio of the formed particles decreased (Supplementary Fig. S3).

**Figure 3.**
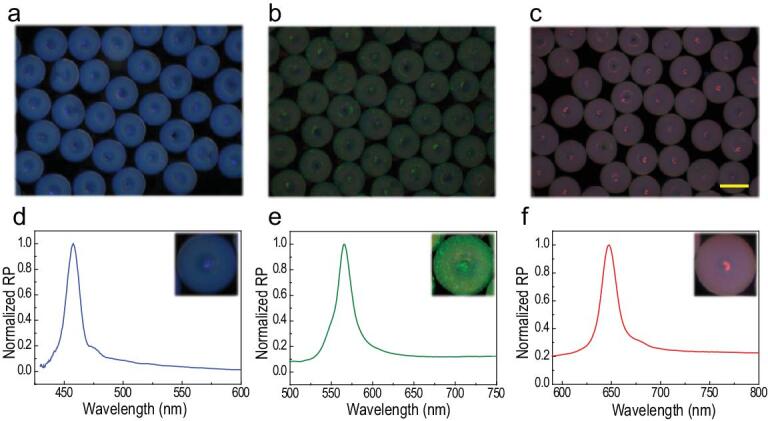
Optical characterization of stomatocyte PhC barcodes. Reflection microscopic images of blue (a), green (b) and red (c) stomatocyte PhC barcodes. The scale bar is 250 μm. (d–f) Reflection spectra of these three kinds of stomatocyte PhC barcodes.

To further verify the stomatocyte morphology of the particles, the scanning electron microscope (SEM) was employed (Fig. 2c). The SEM images indicated that resultant PhC particles assembled by solvent extraction presented stomatocyte morphology on a relatively large scale. At the nanoscale, the microstructural SEM images of stomatocyte PhC particles showed that the nanoparticles formed a good hexagonal alignment in such a short period. The highly ordered 3D structure imparted the stomatocyte PhC particles with corresponding structural color as well as characteristic reflection peaks as encoding elements. This alignment is the same as that in spherical microparticles; thus, under normal incidence, the reflection peak position *λ* follows the Bragg equation:

$$\begin{equation*}
\lambda = \ 1.633d{n_{{\rm{average}}}},
\end{equation*}$$

where *d* is the distance between two neighboring nanoparticle centers and *n*_average_ refers to the average refractive index of the particles. In this study, the reflective peak position mainly depends on the diameter of the silica nanoparticles monodispersed in the droplets, since the duty ratio of different particles is the same. Thus, by changing the sizes of the assembled silica nanoparticles, stomatocyte PhC particles with different characteristic reflection peaks could be fabricated and used as barcodes. Notably, the stomatocyte PhC barcodes showing distinct structural colors including blue, green and red, respectively, could be obtained by employing silica nanoparticles with diameter of 200, 250 and 285 nm (Fig. 3). Their reflective spectrogram indicated that the reflection peaks of barcodes were located in 458, 565 and 650 nm, respectively. Because their optical properties were derived from intrinsic physical structures, the generated stomatocyte PhC barcodes were stable and free from any fluorescent background as well as photobleaching, indicating that they were ideal candidates as encoded microcarriers for detection.

**Figure 4.**
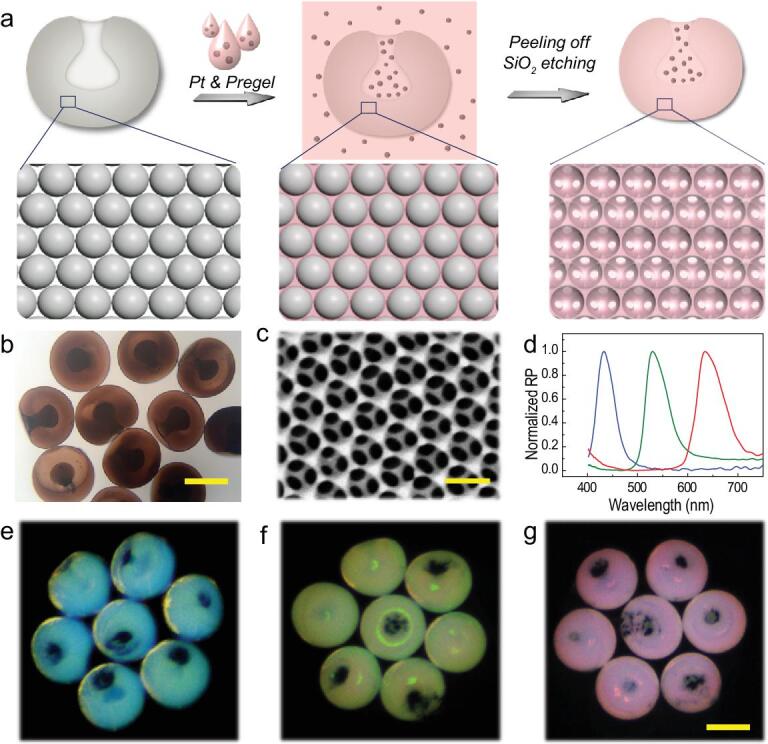
Fabrication of structural color-barcode micromotors. (a) The scheme of the fabrication process. (b) The bright-field microscopic image of the micromotors obtained. The scale bar is 150 μm. (c) The SEM image of the surface nanostructure of the micromotors. The scale bar is 240 nm. (d) Reflection spectra of three kinds of barcodes micromotors. Reflection images of blue (e), green (f) and red (g) barcode micromotors. The scale bar is 160 μm.

Attractively, the stomatocyte PhC barcodes showed their versatilities because their cavities offered the possibility of introducing functional materials. By loading Pt nanoparticles in the cavities, the stomatocyte PhC barcodes could then achieve autonomous movement as micromotors in fluidic environments. To improve their motion abilities, hydrogel was utilized to substitute the relatively heavy silicon nanoparticles while still duplicating the nanostructure of the stomatocyte PhC barcodes (Fig. 4a). In this process, the stomatocyte PhC barcodes were immersed in the hydrogel solution containing Pt nanoparticles first. Owing to the capillary force, the hydrogel solution could enter and fill in the interconnected nanopores among the ordered self-assembly silica nanoparticles. Because their sizes were much bigger than the nanopores, the Pt nanoparticles could only deposit in the cavities. Afterwards, the stomatocyte PhC hydrogel micromotors containing Pt nanoparticles could be achieved by polymerizing under ultraviolet (UV) light, peeling off redundant hydrogel and etching silica nanoparticles by using hydrofluoric acid (Fig. 4b). As the hydrogel micromotors were neg-atively replicated from stomatocyte PhC particles, they had similar highly ordered 3D inverse opal structures (Fig. 4c), which indicated that the hydrogel micromotors were also imparted with characteristic reflection peaks (Fig. 4d) and structural color (Fig. 4e–g) resulting from the PBG. The reflection peaks of the hydrogel barcode micromotors that were blue shifted by about 20 nm due to the change in their average refractive index. These micromotors then gain autonomous movement ability in the environment of H_2_O_2_ and the reflection peaks remains stable while moving (Supplementary Fig. S4). Stomatocyte PhC particles with an aperture ratio of 25% and a diameter of 250 μm were finally chosen for later application due to their stable movement performance. The velocity of the micromotors in different concentrations of H_2_O_2_ were observed and are shown in Supplementary Fig. S5.

**Figure 5.**
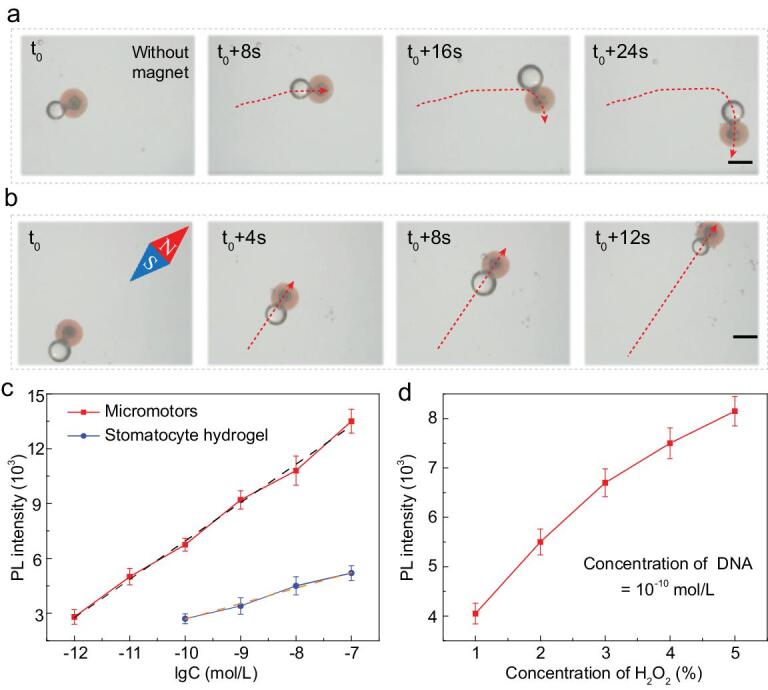
The motion performance of the barcode micromotors and their application in detecting DNA. (a) The time-lapse sequence of the motion route of micromotors without magnet guidance. The scale bar is 250 μm. (b) The time-lapse sequence of the movement of micromotors under magnet guidance. The scale bar is 250 μm. (c) Statistical analysis of the fluorescence intensity at different DNA concentrations (10^−12^ to 10^−7^ mol/L). (d) Statistical analysis of the fluorescence intensity at different H_2_O_2_ concentrations when detecting DNA with a concentration of 10^−10^ mol/L.

Apart from the bubble-driven locomotion, the hydrogel micromotors could also respond to external magnetic fields by dispersing magnetic nanoparticles in the hydrogel solution for duplication. The magnetic nanoparticles could fill the voids in the micromotors with hydrogel, endowing the micromotors with ability of controllable movement under a magnetic field. It can be seen from Supplementary Fig. S6 that the micromotors could be attracted to one side of the container by a magnet, indicating that hydrogel micromotors fabricated using this method exhibited excellent magnetic controllability and could be gathered for reusing. In detail, the movement routes of the micromotors were recorded (Fig. 5a and b). Compared to those without magnetic nanoparticles, which moved randomly in the solution, the magnetic micromotors performed a designed linear trajectory along the guidance of the magnetic field. Also, magnetic micromotors moved at a higher speed due to the magnetic attraction. These features have demonstrated that fabricated stomatocyte barcode micromotors could combine the advantage of striking structural colors of barcodes and their controlled-motion property, which paves new ways for sensing or detection applications.

To investigate their practical value in biosensing application, the structural color-barcode micromotors were employed to detect DNA as examples. The DNA sequences used are listed in Supplementary Table S1. As the ordered porous nanostructure of the hydrogel micromotors enabled efficient probe modification and the autonomous movement increased the probe–target interactions, the probe-modified barcode micromotors could efficiently capture DNA targets in H_2_O_2_ solution. Because of the specific recognition and conjugation of DNA sequences, the targets with fluorescence labels were captured by micromotors modified with corresponding probes and showed the fluorescent signal intensity according to the number of captured target DNA, as schemed in Supplementary Fig. S7. The higher fluorescence intensity of the barcode micromotors than stomatocyte hydrogel after detecting DNA suggested that barcode micromotors could capture more targets because the motion ability of micromotors could efficiently accelerate the mixing speed of the detection sample and thus greatly increase the probe–target interactions (Supplementary Fig. S8). Also, their fluorescence intensities could respond to different concentrations of DNA samples. The statistical results indicated that, by detecting a higher concentration of DNA, the fluorescence intensity of either micromotors or the stomatocyte hydrogel increased logarithmically (Fig. 5c). Notably, the detection limit of micromotors was 7.6 × 10^−13^ mol/L, while that of the normal hydrogel was 8.0 × 10^−11^ mol/L. Additionally, the relationship between the concentration and photoluminescence (PL) intensity was investigated. As H_2_O_2_ with a concentration >5% would affect the PL intensity [19], the speed of micromotors was observed in H_2_O_2_ with a concentration at the range of 1%–5%. It was found that, with a higher concentration of H_2_O_2_, the micromotors achieved a higher motion speed and thus had a more sensitive detection capability (Fig. 5d).

**Figure 6.**
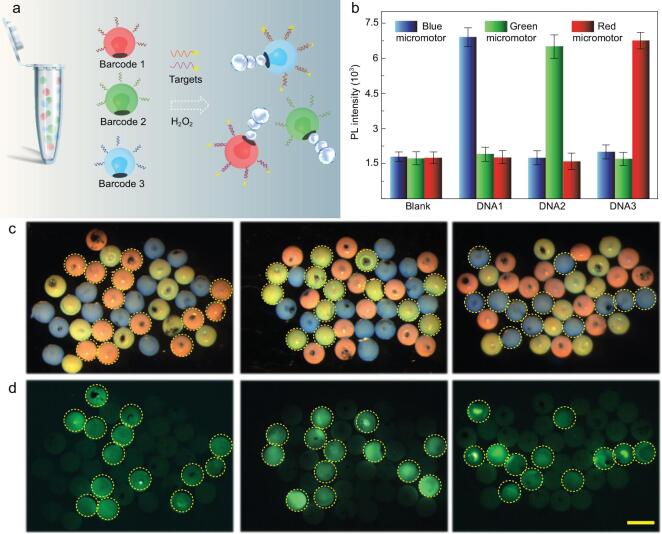
Multiplex detection using structural color-barcode micromotors. (a) The schematic of the nucleic-acid multiplex detection mechanism. (b) The fluorescence statistics after incubating three different barcode micromotors with different targets. Optical microscopy images (c) and fluorescence images (d) of the three kinds of micromotors after incubating with the target DNAs. The scale bar is 350 μm.

Considering that stable structural colors are ideal encoding elements that could be identified specifically, the barcode micromotors could show their privilege in multiplex detection. In order to demonstrate their multiplex analytical performance, structural color-barcode micromotors with characteristic reflection peaks at 458, 565 and 650 nm (appeared blue, green and red) were first modified with Probe1, Probe2 and Probe3, respectively, to simultaneously detect three kinds of DNA. According to the specific recognition of nucleic-acid sequences, fluorescence was expected to be observed if the probe decorated on the barcode micromotors coupled with its corresponding DNA target. Then, the three kinds of micromotors were immersed in H_2_O_2_ solution containing only one kind of labeled DNA target for multiplex detection. By combining the structural color and fluorescence signals, the type of captured target could be easily figured out (Fig. 6). It could be seen that the DNA sequence bonded selectively to the corresponding micromotors and thus the micromotors presented fluorescence signals. Further fluorescence-intensity statistics proved excellent specificity of this detection method and could also be used to indicate the captured DNA quantitatively (Fig. 6b). The detection procedure is simple but sensitive and efficient, suggesting that the fabricated structural color-barcode micromotors are promising candidates for simultaneous multiplexing DNA detection and could be used as ultrasensitive multiplex assays in different fields.

## CONCLUSION

In summary, we presented novel barcode micromotors with distinctive structural colors and characteristic reflection peaks as encoding elements for simultaneously multiplex assays. By the quick solvent extraction and self-assembling of monodispersed silica nanoparticles in aqueous droplets, stomatocyte PhC particles could be fabricated. The particles then exhibit obvious structural colors due to the good hexagonal alignment of the nanoparticles and could be imparted with various functionalities because of the cavity morphology. Therefore, by employing hydrogel dispersed with Fe_3_O_4_ nanoparticles to replicate the highly ordered structure and Pt nanoparticles to fill the cavity, structural color micromotors driven by magnetic force and bubbles generated from the decomposition of hydrogen peroxide could be fabricated. These kinetic characteristics could thus facilitate the detection process. Benefiting from the specific identification of the structural color, the barcode micromotors performed highly sensitive and very flexible detection ability, even in the multiplex DNA detection process. All of the results manifested that structural color-barcode micromotors would significantly promote the development of applications in biosensing analysis, clinical diagnosis and related research areas.

## METHODS

### Materials

The silica nanoparticles with different diameters were self-prepared. The glyceryl triacetate, *n*-butyl alcohol and cyclopropylene glycol and hydrofluoric acid (HF) were provided by Sinopharm Chemical Reagent Co., Ltd.. Acrylic acid (AA), poly (ethylene glycol) diacrylate (PEGDA) with a molecular weight of 700 and 2-hydroxy-2-methylpropiophenone (HMPP) and 1-(3-dimethylaminopropyl)-3-ethylcarbodiimide hydrochloride (EDC) were purchased from Sigma-Aldrich, Shanghai, China. The magnetic nanoparticles were purchased from Nanjing Nanoeast Biotech Co., Ltd. and the platinum nanoparticles were purchased from Aladdin Industrial Corporation. The DNA sequences in this study were obtained from Nanjing Ruizhen Biotechnology Co., Ltd.. Epoxy chloropropane (ECH), tetraethylorthosilicate (TEOS) and N-hydroxysuccinimide (NHS) were purchased from Aladdin Industrial Corporation, Shanghai, China. Phosphate buffer saline (PBS, 0.05 M, pH 7.4) and the 2-morpholinoethanesulfonic acid buffer (MES) were self-prepared. All buffers were self-prepared using water purified in a Milli-Q system (Millipore, Bedford, USA).

### Synthesis of silica nanoparticles

Silica nanoparticles were synthesized using the Stöber method. First, TEOS was added dropwise into the solution mixture of ethanol (300 ml) and ammonium hydroxide (10 ml). With the hydrolysis and condensation reactions of TEOS, silica nanoparticles grow continuously in 30°C with stirring (300 rpm). The nanoparticles were sampled every half hour to get the desired particles with different sizes.

### Fabrication of stomatocyte PhC particles

To fabricate the stomatocyte PhC particles, the silica nanoparticle aqueous droplets with concentrations ranging from 20% to 80% were exposed to an organic extraction in a vertical channel. The volume of the droplets was 0.1 μL. To explore the influence of different extraction agents, glyceryl triacetate, *n*-butyl alcohol and cyclopropylene glycol were utilized as the organic phase, respectively. After the organic phase removed the water in droplets, the stomatocyte PhC barcodes were finally obtained by calcining at 800°C for 12 hours in a muffle furnace to stabilize the structure.

### Preparation of magnet hydrogel barcode micromotors

The magnet hydrogel barcode micromotors were prepared by taking the former fabricated stomatocyte PhC particles as templates. First, the dried stomatocyte PhC particles with brilliant structural colors and characteristic reflection peaks were immersed in a mixed solution that contained 20% PEGDA, 10% AA, 5% magnetic nanoparticles solution, 5% Pt nanoparticles and 1% HMPP (volume ratio) for 30 minutes. AA was added for the introduction of carboxyl, and HMPP was the photoinitiator, which controls the process of polymerization. The concentration of the magnetic nanoparticles with a diameter of about 10 nm was 4 mg/mL. The diameter of the Pt nanoparticles used was <1 μm. After the hydrogel solution had filled the nanopores, ultraviolet light was utilized to polymerize the mixed solution. Subsequently, the polymerized hydrogel on the surface of the templates was mechanically removed by rubbing softly with fingers. The magnetic barcode micromotors were finally obtained by using HF to etch the silica nanoparticles in the templates.

### Preparation of DNA-conjugated hydrogel barcode micromotors

Three kinds of DNA probes were dissolved in sterile water at a concentration of 10^−4^ mol/L. First, the hydrogel micromotors prepared were treated with the MES solution containing EDC and NHS for 30 minutes at 37°C in a constant shaker to realize the activation of carboxyl for better modification of the amino-modified probes. Afterwards, the redundant probes were washed away. Then, by incubating in the PBS with DNA probes for 6 hours at 25°C, the blue micromotors were coated with amino-modified DNA1, the green ones with amino-modified DNA2 and the red ones with amino-modified DNA3. Finally, the different functionalized micromotors with their own probes were prepared successfully after being washed using the buffer solution.

### Capture of DNA

For exploring the detection limit, the barcode micromotors modified with Probe1 were immersed in a 5% (volume ratio) H_2_O_2_ solution containing different concentrations of the target DNA and a fixed concentration of fluorescence labels. The concentration of the target DNA ranged from 10^−13^ to 10^−8^ mol/L and the FAM-decorated label remained at a constant concentration of 10^−6^ mol/L. After 1 hour of incubation, PBS was employed three times to wash away the redundant labels. Finally, the result of the capture was determined by measuring the fluorescence intensity of the micromotors under a fluorescence microscope. Each fluorescence-intensity statistic was measured six times.

For multiplex assays, our barcode micromotors coated with Probe1, Probe2 and Probe3 were mixed and incubated in the solution with only one target DNA and the corresponding label. After 1 hour of incubation, PBS was employed to wash away the redundant labels. Finally, the result of the multiplex detection was determined by comparing the bright-field images and fluorescence images under a microscope.

### Characterization

The forming process of stomatocyte PhC barcodes was observed using a fast camera (F032B, Pike, Germany). Optical images were captured using a stereomicroscope (JSZ6S, Jiangnan novel optics) equipped with a charge coupled device (CCD) camera (Oplenic digital camera). The cross-sectional microstructure of the microspring was characterized using a field emission SEM (FESEM,  Ultra Plus, Zeiss). The fluorescence images of the barcode micromotors were observed using a microscope (Olympus IX71) and recorded using a CCD (Olympus DP30BW). The fluorescence intensity was measured using a fiber-optic spectrometer (Halogen Light Source).

## Supplementary Material

nwz185_Supplemental_Files
